# Evaluating the safety and efficacy of mesenchymal stem cell-derived exosomes for treatment of refractory perianal fistula in IBD patients: clinical trial phase I

**DOI:** 10.1093/gastro/goac075

**Published:** 2022-12-09

**Authors:** Hojjatollah Nazari, Foroogh Alborzi, Asieh Heirani-Tabasi, Alireza Hadizadeh, Reza Akbari Asbagh, Behnam Behboudi, Mohammad Sadegh Fazeli, Mojgan Rahimi, Mohammad Reza Keramati, Amir Keshvari, Alireza Kazemeini, Masoud Soleimani, Seyed Mohsen Ahmadi Tafti

**Affiliations:** Department of Colorectal Surgery, Colorectal Research Center, Imam Khomeini Hospital Complex, Tehran University of Medical Sciences, Tehran, Iran; Department of Biomedical Engineering, School of Biomedical Engineering, University of Technology Sydney, Sydney, NSW, Australia; Department of Colorectal Surgery, Colorectal Research Center, Imam Khomeini Hospital Complex, Tehran University of Medical Sciences, Tehran, Iran; Department of Gastroenterology, Division of Gastroenterology, Imam Khomeini Hospital, School of Medicine, Tehran University of Medical Sciences, Tehran, Iran; Department of Colorectal Surgery, Colorectal Research Center, Imam Khomeini Hospital Complex, Tehran University of Medical Sciences, Tehran, Iran; Department of Cardiovascular Surgery, Research Center for Advanced Technologies in Cardiovascular Medicine, Cardiovascular Research Institute, Tehran University of Medical Sciences, Tehran, Iran; Department of Cardiovascular Surgery, Research Center for Advanced Technologies in Cardiovascular Medicine, Cardiovascular Research Institute, Tehran University of Medical Sciences, Tehran, Iran; Department of Colorectal Surgery, Colorectal Research Center, Imam Khomeini Hospital Complex, Tehran University of Medical Sciences, Tehran, Iran; Department of Colorectal Surgery, Colorectal Research Center, Imam Khomeini Hospital Complex, Tehran University of Medical Sciences, Tehran, Iran; Division of Colorectal Surgery, Department of Surgery, Tehran University of Medical Sciences, Tehran, Iran; Department of Colorectal Surgery, Colorectal Research Center, Imam Khomeini Hospital Complex, Tehran University of Medical Sciences, Tehran, Iran; Division of Colorectal Surgery, Department of Surgery, Tehran University of Medical Sciences, Tehran, Iran; Department of Colorectal Surgery, Colorectal Research Center, Imam Khomeini Hospital Complex, Tehran University of Medical Sciences, Tehran, Iran; Division of Colorectal Surgery, Department of Surgery, Tehran University of Medical Sciences, Tehran, Iran; Department of Colorectal Surgery, Colorectal Research Center, Imam Khomeini Hospital Complex, Tehran University of Medical Sciences, Tehran, Iran; Division of Colorectal Surgery, Department of Surgery, Tehran University of Medical Sciences, Tehran, Iran; Department of Colorectal Surgery, Colorectal Research Center, Imam Khomeini Hospital Complex, Tehran University of Medical Sciences, Tehran, Iran; Division of Colorectal Surgery, Department of Surgery, Tehran University of Medical Sciences, Tehran, Iran; Department of Colorectal Surgery, Colorectal Research Center, Imam Khomeini Hospital Complex, Tehran University of Medical Sciences, Tehran, Iran; Division of Colorectal Surgery, Department of Surgery, Tehran University of Medical Sciences, Tehran, Iran; Department of Cell Therapy and Hematology, Faculty of Medical Sciences, Tarbiat Modares University, Tehran, Iran; Department of Tissue Engineering and Applied Cell Sciences, School of Advanced Technologies in Medicine, Student Research Committee, Shahid Beheshti University of Medical Sciences, Tehran, Iran; Department of Colorectal Surgery, Colorectal Research Center, Imam Khomeini Hospital Complex, Tehran University of Medical Sciences, Tehran, Iran; Division of Colorectal Surgery, Department of Surgery, Tehran University of Medical Sciences, Tehran, Iran

**Keywords:** IBD, exosome, Crohn’s disease, MSCs, fistula

## Abstract

**Background:**

Exosome administration is a novel medical approach that promises excellent immunomodulatory properties without the conventional side effects of current antitumor necrosis factor drugs and stem cells. This study aimed to assess the safety and efficacy of using mesenchymal stem cell (MSC) exosomes to treat refractory fistulas in patients with inflammatory bowel disease.

**Methods:**

MSCs were derived from the umbilical cords and their exosomes were isolated. Five patients with refractory perianal Crohn’s disease fistulas with a median age of 35 years (range 31–47 years) were enrolled in the study. Exosome injections were administered in the operating room to patients with refractory fistula (fistulas that are irresponsive to anti-tumor necrosis factor-α administration within 6 months). Six months later, a physical examination, face-to-face interviews, and magnetic resonance imaging were employed to evaluate the therapy responses of patients.

**Results:**

The outcomes within 6 months after initiation of therapy showed that four patients had responded to therapy. Three patients who received exosome injections exhibited complete healing, while one reported no improvement and active discharge from the fistula site. In addition, five patients (100%) reported neither systemic nor local adverse effects.

**Conclusions:**

Injection of exosomes extracted from MSCs demonstrates safety and a satisfactory therapeutic effect, as evidenced in this and other studies, and may play a significant role in the future treatment of gastrointestinal fistulas.

Core tip

**What this paper adds?**

- In this phase I clinical trial, human subjects with Crohn’s disease and a perianal fistula were treated with MSC-derived exosomes; follow-up magnetic resonance imaging and examination revealed significant healing potential with no adverse side effects or complications.

**How can this study help patient care?**

- Considering the complexity of perianal fistula in Crohn’s disease, the challenging nature of its treatment, and the high recurrence rate in these patients, treatment with more potent and biocompatible methods is essential. Exosome therapy, which has demonstrated high efficacy in immunomodulation as well as great biocompatibility, is a promising new treatment option for many patients with Crohn’s disease-related perianal fistula.

## Introduction

Crohn’s disease (CD) is a chronic immune-mediated inflammatory illness that affects the entire gastrointestinal (GI) tract and is often complicated by intestinal strictures and fistulas. CD-related fistulas can occur between continuous bowel loops or an adjacent organ, such as the bladder, urethra, or vagina. Perianal fistulas occur in 5%–40% of CD cases and are more common in individuals with severe colon and rectum inflammation [1].

Standard surgical interventions for Crohn’s perianal fistula management include abscess drainage and seton replacement; these techniques are primary interventions in acute phase cases with sepsis [2]. Alternative methods for the management of perianal fistula, including fistulotomy, mucosal advancement flaps, ligation of intersphincteric fistula tract, video-assisted anal fistula treatment (VAAFT), and fistula tract laser treatment (FiLaC^®^), are regarded as more effective [3–8]. Due to the anti-inflammatory nature of CD, medical management for perianal fistula closure is common; combined therapy with antitumor necrosis factor-α (TNF-α) (e.g. infliximab) and thiopurines is considered the first-line treatment for Crohn’s perianal fistula [9, 10].

Despite significant advancements in surgical and medical treatment methods, the efficacy of existing approaches involving the combination of anti-tumor necrosis factor (TNF)-α and surgery is estimated to be ≤50%; thus, the optimal approach for Crohn’s perianal complications has yet to be determined [11]. The high failure rate of the current approach can be attributed to multiple factors, including the complex pathophysiology of CD, bacterial overgrowth, infection, and underlying inflammation; these factors pose a serious barrier to effective mucosal healing [12].

Stem cells are a novel alternative to the conventional medical treatments for Crohn’s perianal fistula due to their potential immune-modulatory properties [13]. According to early studies, stem cells can effectively treat Crohn’s perianal fistula [14, 15]. Despite the immunomodulatory effects of stem cells and their significant efficacy in the treatment of perianal fistula, stem cell administration has not been widely adopted due to several safety concerns, including undesirable and uncontrolled differentiation of stem cells, the possibility of malignant transformation and tumorigenicity, and the prospect of acute immunologic reactions [16].

Studies have shown that the stem cells’ immunomodulatory properties are attributed to their extracellular secretions, such as extracellular vesicles and exosomes [17, 18]. Exosomes are lipid bilayer structures that contain bioactive and signaling molecules, including proteins, lipids, and nucleic acids [19–21]. These bilayer lipid vesicles are isolable from blood, urine, saliva, and milk [22]. Small vesicles (30–150 nm) can pass through cell membranes and are biocompatible enough to possess effective contents [23]. The role of exosomes in inflammatory disorders of the GI tract, such as inflammatory bowel disease (IBD), is correlated with signaling pathways and molecules [24–28]. In light of the low efficacy of current medical and surgical treatments, the role of inflammatory pathways in GI tract diseases, and the immunomodulatory properties of stem cell-derived exosomes, it has been proposed that local and systemic administration of exosomes can be a safe and effective treatment for Crohn’s perianal fistula.

This phase I clinical trial aimed to assess the safety of administering exosomes derived from umbilical cord mesenchymal stem cells (MSCs) to patients with complex perianal fistula associated with CD.

## Materials and methods

**Figure 1.**
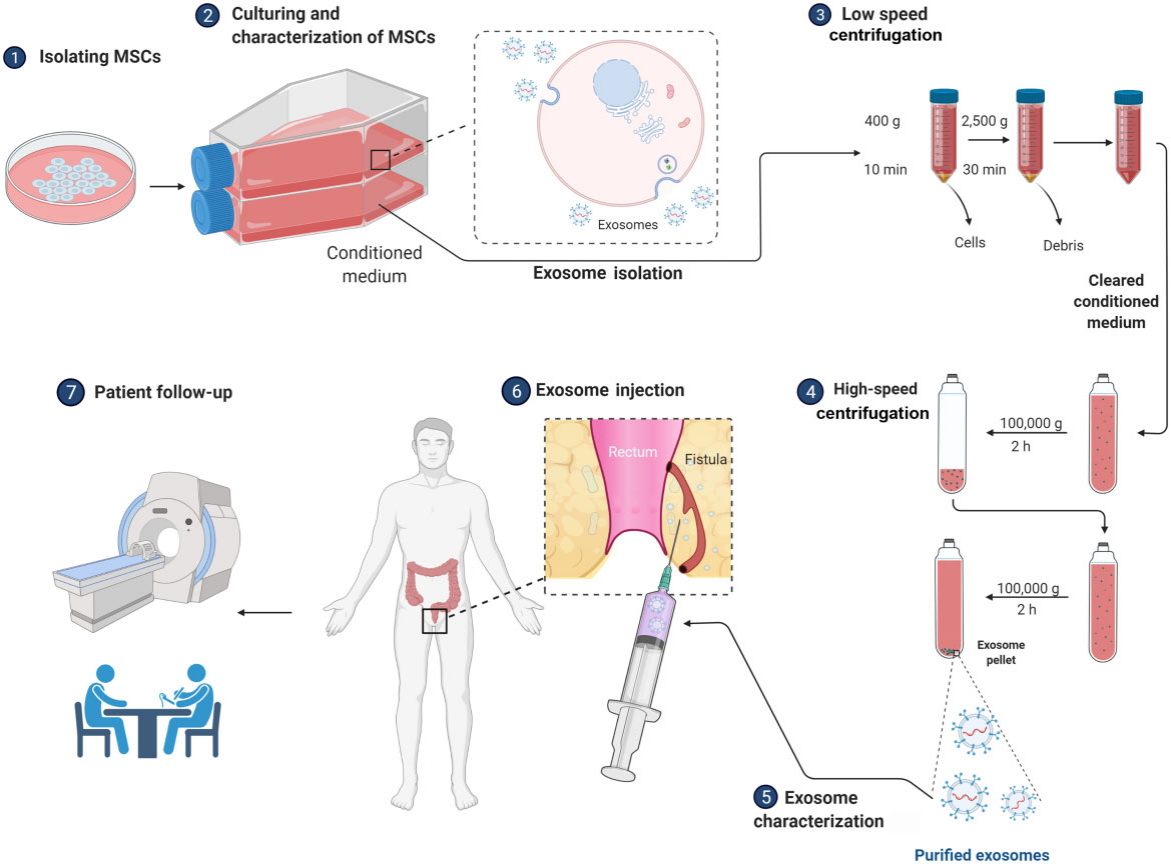
The graphical abstract summarizing the entire process of exosome extraction and clinical aspect of the study

Figure 1 is a schematic representation that illustrates the process of MSC culture, exosomes extraction, and the clinical phase of the study.

### Human umbilical cord MSC isolation and characterization

Human umbilical cords were obtained in accordance with the Imam Khomeini Hospital’s research protocols and the ethical code IR.TUMS.IKHC.REC.1400.184. The cords were placed in phosphate buffer saline (PBS) containing 100 U penicillin/streptomycin (Pen/Strep; Gibco). After removing the blood vessels, the samples were cut into 2- to 6-mm^3^ pieces, washed with PBS, and treated for 2 hours at 37°C with 2 mg/mL of type IV collagenase. The sample was then washed with PBS and filtered through a 70-μm nylon mesh to remove particles and aggregations. The isolated cells were suspended and seeded in the flasks in a solution of D-MEM F12 culture medium (Gibco) supplemented with Pen/Strep and 10% fetal bovine serum (FBS; Gibco). Afterward, the cells were placed in an incubator at 37°C with a 5% CO_2_ concentration and 90% humidity. The exosomes were extracted during the second and third passages. The adipogenic and osteogenic differentiation capacities of MSCs were also investigated and evaluated using a previously defined protocol.

### Exosome isolation and characterization

Third-passage MSCs were cultured in T175 flasks with D-MEM F12 culture medium (Gibco) supplemented with Pen/Strep and 10% exosome-depleted FBS (Gibco) for 48 h at 37°C with a CO_2_ concentration of 5% and 90% humidity. The conditioned media (CM) of MSCs were then collected and centrifuged for 10 min at 400 *g* to remove suspended cells, 30 min at 2,500 *g* to remove debris and apoptotic bodies, and subsequently ultracentrifuged for 120 min at 100,000 *g* (Beckman, USA). Afterward, the supernatants were collected and ultracentrifuged at 100,000 *g* for an additional 120 min. The exosome-containing pellets were then dissolved in PBS.

The protein content of 100 mL of CM vesicles was determined using the Bradford colorimetric assay to confirm the production of MSC-derived exosomes. Dynamic light scattering was employed to determine the size distribution of MSC-derived exosomes. In addition, flow cytometry and Western blotting were used to confirm the expression of CD9, CD63, and CD81. Fluorescein isothiocyanate (FITC) mouse anti-CD63 (BD Pharmingen) and phycoerythrin (PE) mouse anti-CD81 (BD Pharmingen) were utilized as reagents. Furthermore, the morphology and size of isolated exosomes were evaluated using transmission electron microscopy (TEM) according to a previously established protocol. As per a similar study, the optimal volume and dose for maximum effect were calculated to be 5 mL of a 50-μg/mL solution [29].

### Patient characteristics

Ethical approval (IR.TUMS.IKHC.REC.1400) for an open-label, phase Ι prospective clinical trial study of five patients was obtained from the ethical committee of Imam Khomeini Hospital Complex (affiliated with Tehran University of Medical Sciences). Inclusion criteria included (i) patients with refractory disease, (ii) patients previously treated for unhealed fistulas, and (iii) patients who did not improve after receiving 10 mg/kg of intravenous infliximab and seton placement within 6 months. Patients were excluded if they had received anti-TNF-α therapy for <6 months, had a history of cancer or immunodeficiency diseases, or were pregnant.

After obtaining informed consent, patients underwent a general examination and serologic evaluations, including a complete blood count and electrolytes. In addition, the external orifice and fistula tract were investigated while the patients were under general anesthesia.

Conventional multiplanar, multisequence pelvic magnetic resonance imaging (MRI) for perianal fistula detection and characterization was performed prior to MSC-derived exosome use (baseline). The fistulas were classified according to the Park’s classification system. The structure and cavity of the fistula, as well as its extension and T2 hyperintensity, were evaluated. Moreover, the length of hyperintense T2 tracts was determined as a quantitative indicator of fistula activity.

### Exosomes administration program

The procedure was performed in the operating room and patients were under “nothing by mouth” (NPO) orders for 6 hours before the procedure while receiving intravenous sedation and oxygen supplementation through a mask. During the procedure, the patients were positioned for lithotomy. The external fistula opening was examined, the tract was palpated, and an Eisenhammer Retractor was utilized to expose the internal orifice. The tract was irrigated with saline several times using a small catheter to remove pus and fecal matter. A flexible fistula probe was inserted into the tract after irrigation. The tissue surrounding the tract was injected with 5 mL of an exosome solution using the probe as a guide (Supplementary video). The injection depth of the soft tissue and sphincters of the anus was ∼2–3 mm. After the injection, the tract probe was removed and the patients were observed for 3 h in the recovery room while their vital signs were monitored. The patients were then moved to the surgery ward, where they were monitored for 48 h. Six months after injection, the patients were examined under sedation in the operating room for further evaluation.

### Safety evaluation

The central objective of this study was to evaluate the safety of exosomes derived from MSCs for the treatment of IBD-related perianal fistula. Before being discharged from the hospital, patients were observed for 48 h post-operatively to investigate acute adverse effects. In the first 48 h following exosome administration, patients were monitored for ventricular tachycardia, myocardial infarction, stroke, shortness of breath, coughing, and wheezing. In addition, within the first 48 h, patients were monitored for acute allergic reactions, such as hot flushes, urticaria, and skin rashes. Every 6 h, laboratory tests, including complete blood count, liver function tests, erythrocyte sedimentation rate, creatinine, and blood urea nitrogen, were analysed. Patients with no signs of an adverse reaction and normal blood test results were discharged after 48 h. In addition to blood tests and weekly office visits, patients were inspected for side effects and monitored for adverse reactions. GI outcomes such as nausea, vomiting, diarrhea, and abdominal cramping were observed monthly for 6 months.

### Efficacy evaluation

The initial evaluation was conducted within the first month after surgery, with subsequent assessments performed at 4-week intervals until the sixth post-operative month. On each assessment, a comprehensive clinical examination was performed to evaluate any adverse reaction or complication related to the therapy and to assess the fistula tract’s internal and external orifices for healing. Clinical healing was categorized as fully healed (no discharge along with re-epithelialization of the external orifice), relative improvement (decreased drainage), and no change. Short-term adverse events occurred within 4 weeks of injection; long-term adverse events occurred between 4 weeks and 6 months after injection.

### Statistical analysis

IBM Corp. SPSS^®^ software (v. 25) was utilized for statistical analysis in this study. Scale variables were expressed as mean and standard deviation, while nominal variables were expressed using frequency. Statistical significance was considered as *P*-value <0.05.

### Ethics statement

This study was submitted to and ethically approved by the Iranian clinical registry system and committee (reference number IRCT20200413047063N3). This study was also approved by the US clinical trial registry (reference number NCT05499156). This study was also approved by the Research Deputy and the Ethics Committee of the Tehran University of Medical Sciences (reference number: IR.TUMS.IKHC.REC.1400.184) and was carried out per the ethical standards outlined in the 1964 Declaration of Helsinki and all subsequent revisions. All participants signed a written informed consent form.

## Results

### MSC and MSC-derived exosomes characterization

**Figure 2.**
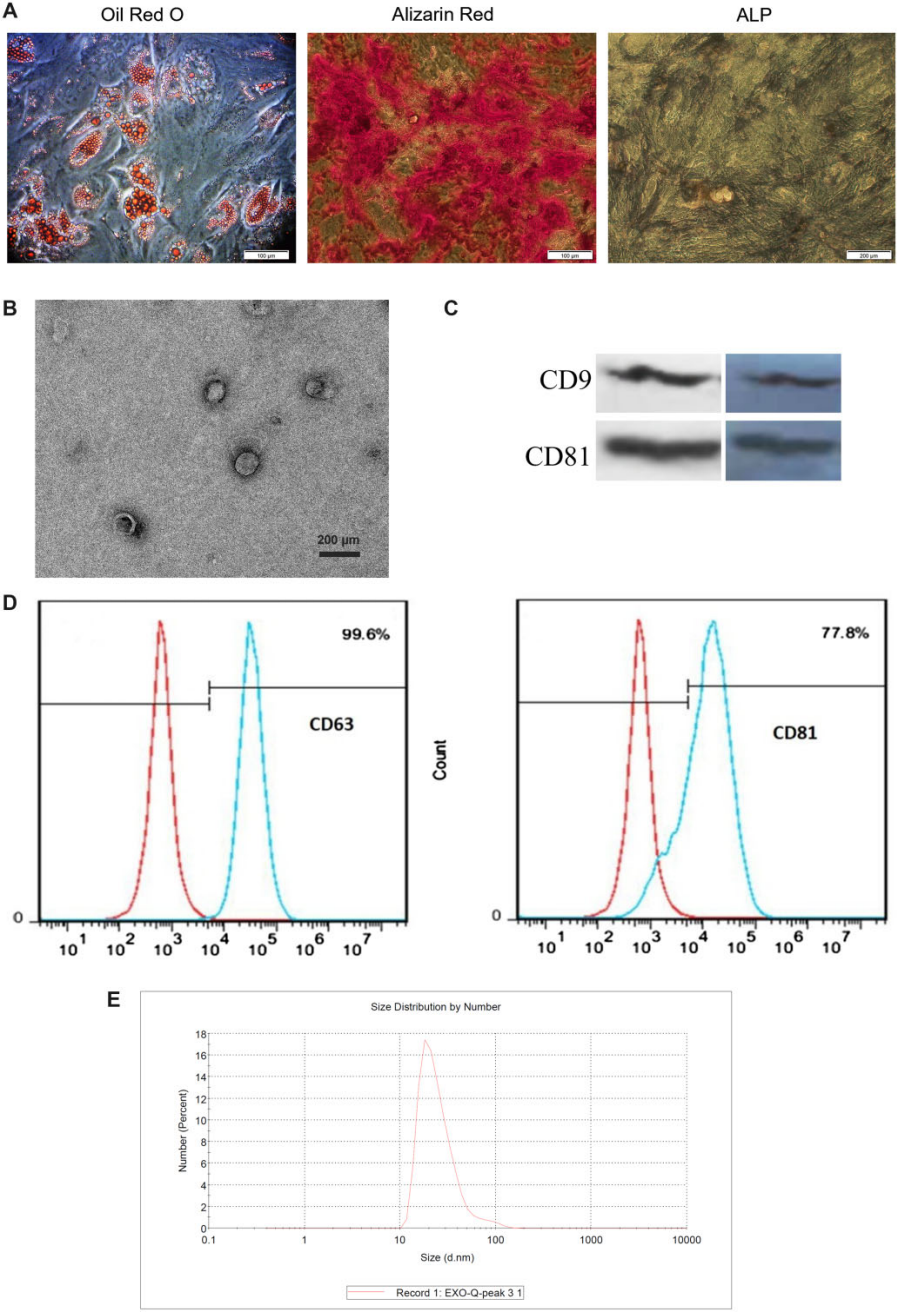
Characterization of mesenchymal stem cells (MSCs) and MSC-derived exosomes. The results of the adipogenic and osteogenic capacity of umbilical cord-derived MSCs (A). The results of exosome characterization via transmission electron microscopy (B), Western blotting (C), flow cytometry (D), and dynamic light scattering (E). ALP, alkaline phosphatase.

The capacity for adipogenic and osteogenic differentiation of umbilical cord-derived MSCs was confirmed through specific staining, Oil red O for adipogenic differentiation, and Alizarin Red and alkaline phosphatase (ALP) for osteogenic differentiation (Figure 2A). TEM was utilized to visualize human umbilical cord MSC-derived exosomes. Consequently, the results demonstrated a spherical shape with a diameter ranging between 30 and 140 nm (Figure 2B).

Western blot and flow cytometry (FC) tests demonstrated high expression of CD63, CD81, and CD9 (Figure 2C and D), consistently with the findings of previous studies regarding exosome size distributions. Exosomes had a diameter of <100 nm, as determined by dynamic light scattering (Figure 2E), consistently with previous research on exosome size distributions.

### Study population

This present trial included five patients with refractory Crohn’s perianal fistula disease. The study population comprised three male and two female subjects with a median age of 35 years (range 31–47 years) (Table 1). The median disease duration at enrollment in the study was 8 years (2–15 years); the median of fistula presence was 24 months (ranging 6–36 months). All patients were previously treated with infliximab for a median duration of 2 years (range 1–3 years). Previously, three patients had their abscesses drained and setons placed. Furthermore, three patients exhibited a single intersphincteric fistula at baseline MRI. One individual had two trans- and intersphincteric fistulae tracts and the Patient 3 had four fistulae, two of which were intersphincteric fistulae tracts. In addition, three patients demonstrated inflammation of the perianal skin surrounding the fistula; all three complained of severe burning, particularly during defecation. One patient experienced difficulty defecating due to extensive fibrotic tissue surrounding the external orifice.

**Table 1.** Patient characteristics prior to exosome therapy

Characteristics	Patient 1	Patient 2	Patient 3	Patient 4	Patient 5
Sex	Male	Male	Female	Male	Female
Age, years	35	47	40	24	31
Disease duration, years	3	8	15	2	13
Clinical manifestation before surgery	Discharge, burning sensation, skin inflammation	Pain, discharge, and pain and difficulty during defecation due to fibrotic ulcer on the internal orifice	Pain, discharge, severe burning sensation particularly during defecation, skin inflammation	Pain, discharge, burning sensation, skin inflammation	Discharge, burning sensation
Number of fistula tracts	2	1	4	1	1
Type of fistula	Two low trans-sphincteric and one intersphincteric perianal fistula	Single intersphincteric perianal fistula	Three intersphincteric perianal fistulae and one trans-sphincteric perianal fistula	Single intersphincteric perianal fistula	Single intersphincteric perianal fistula
Duration of fistula, months	36	36	24	24	6
Duration of anti-TNF-α treatment	1	3	2	2	3
Seton placement	Yes	No	Yes	No	Yes
Crohn’s disease remission status	Silent	Silent	Silent	Silent	Silent
Fistula tract response to therapy	Complete closure	Irresponsive tract persisted but lesser discharge	Three tracts were fully sealed and one trans-sphincteric underwent fistulotomy	Complete closure	Complete closure

TNF, tumor necrosis factor.

### Effects of MSC-derived exosome administration

#### Treatment safety

Patients who received exosomes derived from MSCs did not experience any short- or long-term adverse events. Moreover, no patient complained of adverse outcomes during subsequent evaluations. Laboratory tests revealed no abnormalities after injection or during follow-up; neither leucocytosis nor abnormal liver function tests were detected.

#### Treatment efficacy

**Figure 3.**
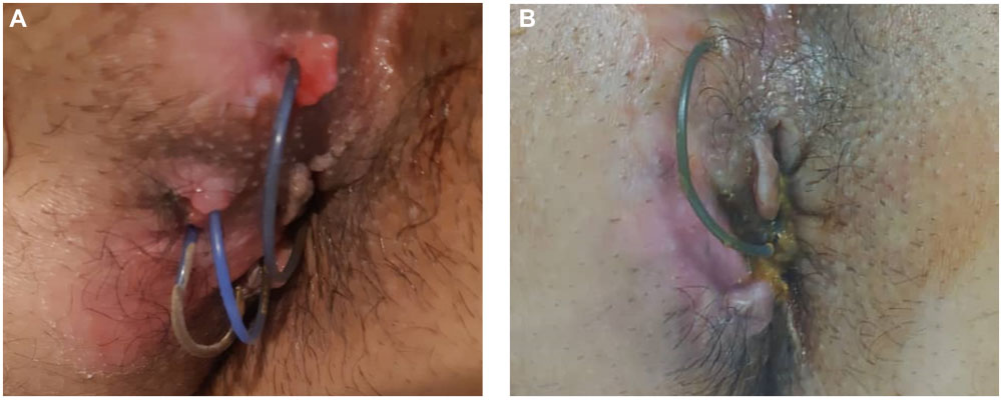
A case (Patient 3) of Crohn’s disease with three perianal fistula tracts. (A) Before exosome therapy; (B) the same patient 6 months after treatment.

**Figure 4.**
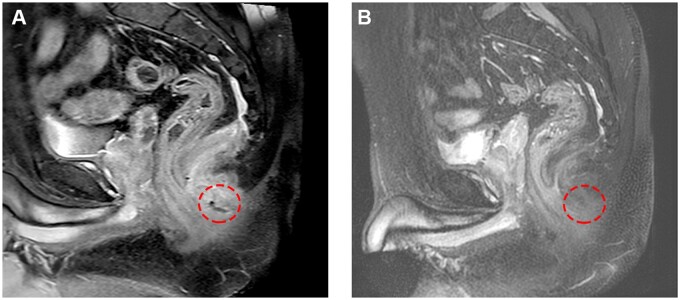
MRI images of a patient (Patient 4) with a fistula before (A) and after (B) 6 months of exosome injection reveal that the tract has been resolved

At the 6-month follow-up, four patients (80%) had responded to treatment; the patient with severe fibrosis around the fistula tract and perianal region had no remarkable outcome, with no change in drainage and re-epithelialization. In two patients with intersphincteric fistula, the fistula was completely closed. The external orifice of the patient with trans-sphincteric fistula completely healed and the patient reported less discharge. The symptoms of the patient with four fistula tracts improved significantly. However, one of the tracts showed signs of re-epithelialization or inflammation. Therefore, the seton was left in place and a simple fistulotomy was performed (Figure 3). In each of the three patients with severe skin inflammation, the skin irritation resolved after 1 month and remained unchanged after 24 weeks. None of the patients reported a burning sensation or pain during the monthly follow-ups. MRI imaging confirmed the closure of fistulae in two patients (Figure 4). In addition, three of the four tracts of the patient with multiple fistulae were completely healed and sealed. The other, in contrast, showed no signs of inflammation. Three months after injection, MRI on two patients revealed persistent tracts (one patient had clinical improvement with less discharge and resolution of skin irritation). Before initiation of exosome therapy, all the patients had small bowel involvement in the ileum. None of the patients had proctitis and the fifth patient had Crohn’s colitis. All Crohn’s patients were inflammatory type, none of them was fibro-stenotic, and none of them had GI fistula. The Patients 1, 3, and 5 had undergone non-cutting drainage seton placement.

## Discussion

Perianal fistula is the most prevalent type of fistula in IBD patients, affecting 20% of patients with CD [30, 31]. Despite significant advancements in medical treatment methods, the current success rate of treatments is estimated to be slightly above 50% [32].

MSCs and their related biomolecules and compounds are promising immunoregulatory therapeutic agents for treating inflammatory disorders. In a 2003 case study, MSCs were used to treat a female patient with an unresponsive rectovaginal fistula caused by CD and unresponsive to a surgical and therapeutic treatment. According to the study, this novel treatment is safe and effective [33]. Due to the anti-inflammatory, immunomodulatory, proliferative, and differentiating properties of MSCs, it has been hypothesized that these cells may be useful in treating IBD fistulas resistant to other treatments.

Early research indicates that MSC-derived exosomes influence multiple signaling pathways, including Zonula Occuldens-1 (ZO-1), annexin-1 (ANXA1), and inteleukin-6 (IL6), TNF-α, mitogen-activated protein kinases, originally called extracellular signal-regulated kinases, and tumor necrosis factor receptor 2 and nuclear factor kappa-light-chain-enhancer of activated B cells (TNFR2/NF-κβ) pathways, which may aid in the healing process of various IBD complications. These exosomes significantly reduce the expression of inflammatory cytokines, including IL-1β and IL-6, while increasing the expression of anti-inflammatory cytokines such as IL-10. Furthermore, extracellular vesicles lower cullin-1 and neural-precursor-cell-expressed developmentally downregulated 8 (NEDD8) levels, linked to neddylation as a post-translational pathway associated with IBD [34]. Furthermore, exosomes reduce the phosphorylation of Janus kinase 1 (JAK1) and signal transducer and activator of transcription 1 (STAT1) in the colon, implying that these bilayer particles modulate immune responses by suppressing JAK1 and STAT1 [35].

This is the first study to assess the safety and effectiveness of exosomes as a treatment for IBD perianal fistula. The results of this study suggest that exosome therapy is a viable method for treating refractory perianal fistulas, as none of the patients developed acute or latent complications and reactions. During the trial, neither laboratory abnormalities nor clinical symptoms were observed. Clinical examinations and MRI scans revealed that four patients (80%) responded to therapy. All patients were cured of skin inflammation, which alleviated burning sensation. However, treatment was ineffective for tracts with fibrosis surrounding the tract and the internal opening. We hypothesize that this occurrence results from the inability of exosomes to penetrate fibrotic tissue. Consequently, this technique is unsuitable for chronic conditions with significant fibrosis. No patient experienced adverse effects or complications during the 6-month follow-up.

Similar studies previously evaluated the efficacy and safety of MSCs as a novel treatment for IBD complications [14, 15]. Molendijk *et al.* demonstrated that using MSCs in treating 21 patients with Crohn’s perianal fistula was safe, with a success rate comparable to the present study (80%) [36]. In another study, Lightner *et al.* [30] evidenced that using MSCs to treat Crohn’s rectovaginal fistula is safe, with a 70% success rate. Lightner *et al.* [30] also demonstrated that MSC-coated plugs are safe and effective in 80% of patients with rectovaginal Crohn’s fistula. Based on the studies above and our findings, we hypothesize that the efficacy of MSC-derived exosomes is comparable to that of MSCs. However, as exosomes are extremely unlikely to induce a negative immune response, their application could be safer and potentially more effective; furthermore, this application eliminates the possibility of unintended cell differentiation that could lead to adverse outcomes observed in MSC applications.

Although similar results were obtained in other studies, the comparison is not fully replicable because the settings in which these studies were conducted are not the same. Furthermore, the presence of multiple fistular tracts in a single patient complicates the comparison. The current study evaluates the efficacy of MSC-derived exosomes for patients with complex perianal fistulas, whereas Lightner *et al.* [30] and Topal *et al.* [14] focused on patients with a single tract and no other concurrent tracts.

As ethical and safety concerns regarding the use of MSCs continue to grow, exosomes derived from MSCs, which presumably pose almost no risk in this regard, are preferable [16]. Concerns regarding the tumorigenicity of MSCs have also increased. There is evidence that undifferentiated human embryonic stem cells (hESCs) can develop into teratoma [37] and that MSCs can differentiate into undesirable tissues such as calcified tissues. Moreover, Kuriyan *et al.* [38] demonstrated that the local *in vivo* microenvironment contains factors that can induce MSCs to differentiate in an undesirable manner. In addition to concerns about unintended differentiation, MSCs may promote tumor development and metastasis. MSCs may also migrate to the tumor site and inhibit the antitumor immune response [39]. In this study, we evidenced that using exosomes for IBD fistulas does not result in serious adverse events during the 6-month follow-up period. However, conclusive evidence does not support safety concerns regarding the application of MSCs. Studies with larger sample sizes and longer follow-up periods are required to evaluate the safety of MSCs further and compare it to the administration of MSCs alone.

In conclusion, MSC-derived exosomes have demonstrated high potency and potential safety for treating perianal fistulas in IBD patients.

## Supplementary Material

goac075_Supplementary_Data
